# Lysophospholipid Signaling in the Epithelial Ovarian Cancer Tumor Microenvironment

**DOI:** 10.3390/cancers10070227

**Published:** 2018-07-09

**Authors:** Yan Xu

**Affiliations:** Department of Obstetrics and Gynecology, Indiana University School of Medicine, 1044W. Walnut Street R4-W037, Indianapolis, IN 46202, USA; xu2@iu.edu

**Keywords:** lipids, lysophospholipids (LPLs), lysophosphatidic acid (LPA), sphingosine-1-phosphate, tumor microenvironment (TME), epithelial ovarian cancer (EOC)

## Abstract

As one of the important cancer hallmarks, metabolism reprogramming, including lipid metabolism alterations, occurs in tumor cells and the tumor microenvironment (TME). It plays an important role in tumorigenesis, progression, and metastasis. Lipids, and several lysophospholipids in particular, are elevated in the blood, ascites, and/or epithelial ovarian cancer (EOC) tissues, making them not only useful biomarkers, but also potential therapeutic targets. While the roles and signaling of these lipids in tumor cells are extensively studied, there is a significant gap in our understanding of their regulations and functions in the context of the microenvironment. This review focuses on the recent study development in several oncolipids, including lysophosphatidic acid and sphingosine-1-phosphate, with emphasis on TME in ovarian cancer.

## 1. Introduction

The tumor microenvironment (TME) for epithelial ovarian cancer (EOC) is rather unique. It is mainly confined within the peritoneal cavity and frequently associated with ascetic fluid [1,2,3,4,5,6]. The TME consists of many stromal cell types including: tumor-associated macrophages (TAMs), T cells (e.g., regulatory T cells), tumor associated fibroblasts (TAFs), mesothelial cells, adipocytes, endothelial cells (ECs), myeloid-derived suppressor cells (MDSCs), pericytes, platelets, extracellular matrix components (EMCs), and cell-free factors [1,2,3,4,5,6].

The presence of ascetic fluid provides a mobile, easy access, and more dynamic environment for tumor–stromal interactions. In addition to tumor and stromal cells, EOC ascites is rich in cell-free inflammatory cytokines, chemokines, matrix metalloproteinases, integrins, and other secreted molecules, including bioactive lipid factors. These factors are generated by and mutually function in both tumor and stromal cells via autocrine/paracrine mechanisms. They may exist in either extracellular vesicles (EVs) or in “free” forms, including bond forms to proteins or other molecules [7]. EVs are membrane surrounded structures released by cells in an evolutionally conserved manner, but their release, contents, and/or up-take may be abnormally regulated in cancer. The major populations include microvesicles (MVs, 100–1000 nm), exosomes (30–100 nm), and apoptotic bodies [8]. Exosomes have emerged as new as diagnostic markers, as well as cell-to-cell communication vehicles, with therapeutic applications [4,9,10]. The compositions of exosomes from different cell types are complex, containing ~200 lipids, >3000 proteins, ~1600 mRNA and ~800 microRNAs [11,12,13].

Metabolic reprogramming is one of the major cancer hallmarks [14] that is critical for cancer cells to adapt to stress from TME and the increased nutritional requirements during their growth. These modifications occur through cross-talk between tumor and stroma cells in TME in a dynamic network that connects different molecular processes, such as energy production, inflammatory response, and drug resistance [15]. In particular, primary EOC is characterized by abnormal lipid metabolism and energy disorders. In addition, recurrent EOC patients have been shown to have increased amino acid and lipid metabolism compared with primary EOC patients [16].

Compared to other major living cell components, including DNA, RNA (all composed by four bases), proteins (all composed of 20 amino acids), and carbohydrates (all have the basic element CH_2_O with ring, chain, and branched structures), lipids are very diverse in both their respective structures and functions. These diverse compounds are grouped into classes; glycerophospholipids (PLs) (including lysophospholipids (LPLs)) sphingolipids, sterol lipids, prenol lipids, saccharolipids, and polyketides [17]. The functional involvement of many of these lipids in EOC, PLs, LPLs, sphingophospholipids (SPLs), fatty acids, cholesterol, vitamins, and triglycerides (TGs) in particular, have been covered by many reviews [3,18,19,20,21,22,23]. This review will focus on recent development of signaling LPLs with an emphasis on TME in EOC.

## 2. LPLs

**Figure 1:**
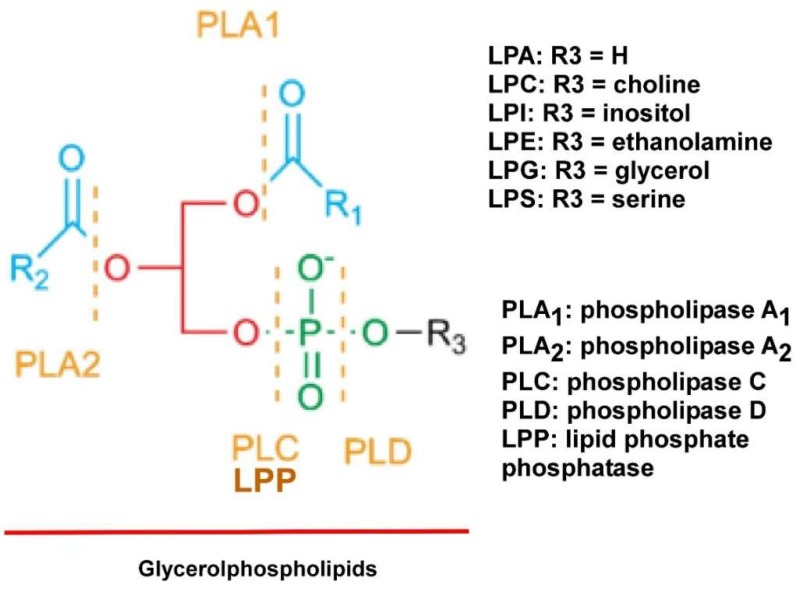
Structures of glycerophospholipids (PLs), lysophospholipids (LPLs only have R_1_ or R_2_) and the action sites of phospholipases.

Compared to PLs, which have two fatty acid chains, LPLs only have one fatty acid chain and thus have reduced hydrophobicity (Figure 1). In addition, many LPLs are either negatively or positively charged, increasing their polarity and solubility in water. With these chemical properties, LPLs are synthesized and/or secreted extracellularly and many of them function as signaling molecules through their specific membrane receptors. In addition, several of these LPLs have tumor promoting activities and are thus termed as “oncolipids” [24]. They are accumulated in the TME. LPA is a prototype of the LPL signaling molecules. It exhibits pleiomorphic functions in almost all cell lineages tested. Since our early report on LPA’s effect in EOC [25,26], more than 1000 papers have been published reporting the roles and signaling mechanisms of LPA in various cancer types [24,27].

### 2.1. LPA

Lysophosphatidic acids (LPAs) are a group of compounds with various fatty acid side chains, differing in their length (commonly 14–22 carbons) and number of double bonds (commonly from zero to six in most tissues). In addition, the chemical linkages of the fatty acid chain to the glycerol backbone can be differently grouped as acyl-, alkyl-, and alkenyl-LPAs [28]. We have originally identified LPA as an EOC growth factor, termed ovarian cancer activating factor [26,29]. Numerous papers have now been published to show that LPA induces a broad range of tumor promoting activities in EOC and LPA is a therapeutic target for EOC [30,31,32].

#### 2.1.1. Increased LPA Levels in EOC

We have initially reported LPA as a potential marker for EOC [33]. Blinded [34] and numerous independent studies have confirmed that LPA is elevated in the blood from EOC patients, when compare to those with benign diseases and/or healthy controls [35,36,37,38,39,40,41,42]. The mean values of physiological and pathologic concentrations of plasma LPA in healthy women and EOC patients are 0.6–0.9 µM and 2.0–22 µM, respectively [33,34]. The mean values of benign and malignant ascites acyl-LPA concentrations are 2.9 µM and 19–95 µM, respectively [28,43]. The mean non-acyl (both alkyl-, and alkenyl)-LPA levels are 3.7 ± 1.7 and 0.9 ± 0.7 µM for benign and malignant ascites, respectively [28] (Table 1). The concentrations of LPA and other lipids mentioned below were measured in cell-free plasma or ascites. In most reports, it is unclear whether these lipids are associated with MVs, as mentioned in the Introduction, in a protein bound, or a “free” form. In an attempt to test this, we have separated different MVs and the cell- and vesicle-free (S4) portion of ascites via step-wise centrifugation. We found that human EOC ascites S4 portion potently promotes proliferation, migration, and invasion of human EOC cells in a PLA_2_-dependent manner, suggesting that LPA, and maybe other LPLs, may present in a protein bound or a “free” form [44]. However, this issue needs to be more systematically investigated and lipid association with different extracellular components may also be cell-type and context dependent.

The challenges to move LPA or other lipid molecules as cancer markers into clinics are several fold. First, as metabolites, these molecules have quick turnover times by their producing and degradation enzymes. In addition, their levels are likely to be affected by other physiological/pathologic conditions—such as diet, smoking, and drinking—which have not been completely investigated. Secondly, due to their chemical properties, the different extraction, storage and detection conditions/methods used in different labs significantly affect the levels detected. However, there are no standard procedures established. Finally, the best detecting method for these lipid markers is electrospray-mass spectrometry (ESI-MS), which is not a routine setting in clinics currently. These challenges have made cross-examination and validation of these markers difficult. Studies to standardize the procedures of collection, extraction, storage, and measurements of lipid marker are critical. Nevertheless, technique advancements are emerging. In particular, mass spectrometry will likely become a routine technique in regular clinic settings in the near future.

#### 2.1.2. LPA Production and Regulation

LPA is produced from secreted enzymes from lysophosphatidylcholine (LPC) by autotaxin (ATX), as well as phospholipase A_2_ (PLA_2_) by either providing the substrate LPC for ATX, or directly produce LPA from phosphatidic acid (PA) [49,50,51,52,53]. LPA is degraded outside cells by a family of three enzymes called the lipid phosphate phosphatases (LPPs) (Figure 1). Imbalanced expression and/or activity levels of ATX, PLA_2_s, and/or LPPs are involved in EOC [54] (see Figure 1 in [54]). The ATX/LPA axis has received increasing interest as a target in cancers, fibrotic diseases, autoimmune diseases, arthritis, chronic hepatitis, obesity, and impaired glucose homeostasis [55]. At least one of the synthetic ATX selective inhibitors is in clinical trials for idiopathic pulmonary disease [55].

EOC cells may produce LPA upon stimulations [50,51,52]. However, the tissues and cells in EOC TME are likely to be the major source of elevated LPA in EOC. The cell types involved in LPA production include, but are not limited to, platelets, adipocytes, mesothelial cells, and immune cells.

Platelet activation generated LPA used to be considered the major source of plasma LPA [56,57]. Paraneoplastic thrombocytosis has been recognized as a prevalent phenomenon in patients with ovarian cancer since the early 1970s [58]. Cancer patients have a ~4-fold increased risk of venous thromboembolism compared with the general population and this is associated with significant morbidity and mortality [59,60]. The preventative and therapeutic significance of blocking thrombopoietic factors (cytokines and lipids) have been noted as an interesting direction in EOC research [60,61,62]. Tumor cells hijack platelet functions by activating them though platelet aggregation [63]. Activated platelets may help tumor cells survive immune surveillance by acting as protective “cloaks” against immune destruction. However, the major tumor cell beneficiary activities are likely mediated by the factors secreted from platelets after activation. These factors include cytokines (such as interleukin-6 (IL-6)), TGF-β, and lipid factors, which mediate the inflammatory, proliferative, and proangiogenic activities of platelets to promote tumor growth, tissue invasion, and metastasis [63]. Increased platelet counts and platelet activation associated with EOC are likely to be important contributors for the elevated LPA levels in EOC TME. In addition, LPA promotes platelet aggregation [64,65] and blocking platelet function leads to inhibition of metastasis of breast cancer through decreased LPA signaling. ATX is detected in platelet α-granules. Functionally active ATX is eventually released following tumor cell-induced platelet aggregation, thereby promoting metastasis [66].

EOC cells preferentially metastasize to omentum, the adipose tissue, which secretes many chemotactic cytokines and growth factors, including LPA [67,68,69]. Adipocytes promote ovarian cancer metastasis and provide energy for rapid tumor growth [70]. Since the identification of ATX as the major LPA producing enzyme [21,71] and studies conducted using mouse ATX knockout models, it has become clear that adipose tissue are at least one of the major tissues in EOC TME that produces LPA [72,73]. Approximately 40% of body ATX is produced by adipocytes, and this is further increased by inflammation [74,75]. The use of ATX inhibitors seems an attractive strategy to produce novel medicinal agents, for example anticancer agents [55].

EOC cells that metastasize within the peritoneal cavity wall and the organs enclosed are coved by a layer of peritoneal mesothelial cells. We have shown that human peritoneal mesothelial cells constitutively produce LPA, which accounts for a significant portion of the chemotactic activity of the conditioned medium from peritoneal mesothelial cells to ovarian cancer cells [76]. The calcium-independent phospholipase A_2_ (iPLA_2_), and cytosolic PLA_2_ (cPLA_2_) are involved in this production and LPA’s tumor promoting activities [76].

Although many types of immune and endothelial cells are involved in EOC TME, their contributions to LPA production and/or degradation are less known. Steady-state ATX is expressed by only a few tissues, including high endothelial venules in lymph nodes, but inflammatory signals (enriched in EOC TME) can upregulate ATX expression in different tissues [77]. In addition, when ECs are con-cultured with EOC cells, coherent and non-cell line specific changes in fatty acids, glycerophospholipids, and carbohydrates, induced by endothelial cell contact are observed over time [78]. Wong and Reinartz et al. have reported that macrophage-derived phospholipase PLA_2_G7, which may produce extracellular LPA, is involved in EOC and associated with early relapse of EOC. It is a secreted enzyme that may produce LPA and arachidonic acid [79,80].

Mice with homozygous deletion of LPP1 (a LPA degradation enzyme) in stromal cells result in elevated levels and decreased turnover of LPA in vivo. In turn, enhanced tumor seeding in the LPP1 KO mice compared to wild type was observed [81].

Taken together, the host cells play an important role in producing and degradation LPA, which may be present in cell free forms in either exosome and/or EV-free forms [44].

#### 2.1.3. Major Cellular Functions and Signaling Mechanisms of LPA in EOC

LPA stimulates almost every aspects of tumor promoting activities, including cell proliferation or differentiation, prevents apoptosis induced by stress or stimuli, induces platelet aggregation stimulates cell morphology changes, cell adhesion, cell migration, and cell invasion. It also stimulates tumorigenesis and metastasis in vivo [25,26,30,31,32,50,51,76,82,83,84,85,86,87,88,89,90,91,92,93,94,95,96,97,98,99].

LPA regulates many pro-tumorigenic and pro-inflammatory factors, including vascular endothelial growth factor (VEGF), matrix metalloproteinases (MMPs), urokinase plasminogen activator, IL-6, IL-8, CXC motif chemokine ligand 12/CXC receptor 4, COX2, cyclin D1, Hippo–YAP, and growth-regulated oncogene alpha. These regulations are at the transcriptional, translational, and epigenetic levels [19,34,44,51,76,82,83,84,86,88,89,90,91,94,95,100,101,102,103,104,105]. LPA induces loss of junctional β-catenin, stimulates clustering of β1 integrins, and enhances the conformationally active population of surface β1 integrins. Furthermore, LPA treatment initiates nuclear translocation of β-catenin and transcriptional activation of Wnt/β-catenin target genes, resulting in gain of mesenchymal marker expression [106]. Gglycodelin, a glycoprotein, is over-expressed in various malignancies, including EOC, and its expression correlates with the diagnosis and prognosis of cancer patients. The expression of glycodelin can be regulated by stromal cells and LPA [107]. While LPA’s function and signaling in EOC has been rather extensively reviewed [19,100,108], several notions and recent developments are specifically noted here.

Firstly, LPA is a confirmed mitogen in many cell types. However, MTT dye reduction is not a good method to measure this effect. LPA affected MTT dye reduction with an unknown mechanism in EOC cells [109], making it an unreliable indicator for cell number changes. In addition, MTT dye reduction may not be sensitive enough to detect DNA replication as [^3^H]tymidine incorporation [25,26,29]. 

Secondly, the most potent roles of LPA in EOC and other cancer cells are likely to be cell migration and invasion. This action is mediated by LPARs and G_i_ and G_12/13_ [30,51,76,83,89,95,104]. This is correlated to LPA’s in vivo effects, where LPA mainly stimulates metastasis, instead of primary tumor growth [89,95]. In comparison, EGF and other growth factors are likely to be more effective in cell proliferation than LPA, but the latter is more effective in induction of cell migration and invasion [30,51,89,90,93,95].

Thirdly, LPA has been recently shown to be involved in cancer stem cells (CSC) in EOC [84,110]. Seo et al. have shown that EOC CSC produces LPA, which augments CSC characteristics such as sphere-forming ability, resistance to anticancer drugs, tumorigenic potential in xenograft transplantation, and high expression of CSC-associated genes, including OCT4, SOX2, and aldehyde dehydrogenase 1 (ALDH1). These actions are mediated by LPAR_1_. ATX is highly secreted from ovarian CSCs. Inhibition or knockdown of ATX markedly attenuates the LPA-producing, tumorigenic, and drug resistance potentials of CSCs. In addition, clinicopathological analysis shows a significant survival disadvantage of patients with positive staining of ATX. In addition, LPA is involved in the crosstalk between CSC in TME. EOC cells secrete LPA that activates the expression and secretion of CXCL12 by mesenchymal stem cells (MSCs), enhancing the resistance of OVCA cells to hyperthermia [23].

Fourthly, the majority of LPA signaling is mediated by its six G protein couples receptors (GPCRs) [100,111]. Among them, LPAR_1–3_ belong to the endothelial differentiation gene (Edg) family of GPCR and LPAR_4–6_ belong to the purinergic P2Y family of GPCRs [111,112]. While LPAR_1–3_ in general mediate LPA’s tumor promoting activities [76,113,114], limited reports showed that LPAR_1_ may represent a negative regulatory LPA receptor inducing apoptosis in ovarian cancer cells [96]. At least three compounds blocking these receptors have passed phase I and phase II clinical trials [115]. Compared to LPAR_1–3_, LPAR_4–6_ are less studied. Both pro- and anticancer effects mediated by LPA_4–6_ in various cancers have been reported, with the majority of them reporting anti-cancer effects [116,117,118].

Finally, LPA has also been identified as a ligand for the nuclear receptor peroxisome proliferator-activated receptor gamma (PPARγ) [119,120]. However, the LPA-PPARγ studies are mainly limited to the vascular and metabolic processes [121,122]. The roles of PPARγ-mediated LPA effects in cancer are essentially unknown until recently. Emerging evidence, however, suggest that this is an important missing opportunity in cancer research. We have recently shown that LPA dose- and time-dependently upregulated SOX9 in EOC cells. This upregulation is mediated by PPARγ. SOX9 was involved in cellular activities related to Cancer Stem Cells (CSC), including anokis-resistance, regulation of CSC marker CD44, and spheroid-formation [85]. In addition, we have shown that LPA effectively upregulates ZIP4 (a zinc transporter) expression via by PPARγ and LPA’s promoting effects in CSC-related activities in HGSOC cells is at least partially mediated by ZIP4 in an extracellular zinc-independent manner [84]. These findings emphasize the importance of targeting by PPARγ for LPA’s tumorigenic actions.

#### 2.1.4. LPA in the Immune System

The ATX-LPA axis has emerged as a novel regulator of lymphocyte homing and inflammation. LPARs are expressed by T cells and LPA enhances the motility of human and mouse T cells in vitro, although generally not in a direct manner [77]. Cancer cells must evade the immune system during metastasis. LPA facilitates this important process by inhibiting CD8^+^ T cell activation [75]. LPA also regulates macrophage differentiation and T cell motility [123,124]. Although EOC cells mainly express the LPAR_1–3_, LPAR_6_ is the main LPA receptor on TAM and tumor-associated T cells [123].

ATX is expressed by lymphoid organ high endothelial venule. LPARs receptors are expressed by NK cells, mast cells, eosinophils, and B cells [77]. In addition, tumor-associated macrophages (TAMs) produce LPA [123]. However, how LPA signaling in stroma cells, and in immune cells in EOC in particular, remains to be further investigated.

In summary, LPA, a simple molecule that mediates a plethora of biological effects, may be targeted at its levels of production by ATX or PLA_2_s, LPA receptors, including PPARγ, or through LPA degradation by lipid phosphate phosphatases (LPPs). The targeting strategy should take TME into serious consideration. Drugs for these applications have been and will soon be entering clinical practice [27].

### 2.2. LPC

Compared to LPA, plasma LPC levels are usually 10 to 100-fold higher and are in the 100–200 µM range in human subjects [34] (Table 1). LPC levels have been shown to be significantly elevated in the plasma of ovarian cancer patients [34,125,126]. On the other hand, others and we have shown that patients with malignant cancer diseases have attenuated LPC plasma levels [127,128,129,130,131]. Moreover, different phospholipase A_2_ enzymes, which mainly convert phosphatidylcholine (PC) to LPC have been shown to be functionally involved in EOC and/or as markers for various cancer types [7,44,50,51,91,126,132,133].

LPC is present at the highest concentrations among LPLs. Its role in signing is still debatable. Although both of its tumor-promoting and suppressing activities have been reported in various cancers [96,129,134,135], specific attention should be paid that LPC is not present in a free form in most physiological and pathologic conditions. It binds to albumin and other carrier proteins [136,137]. The bound form of LPC may not have many of the effects reported previously [91,137]. In particular, when high concentrations (>20 µM) of free LPC are used, it may have non-specific and detergent-like effects, which are unlikely to be physiologically or pathologically significant. LPC may function as a component of cell membrane and carrier to deliver choline to tissues and LPC is the precursor/substrate for ATX to produce LPA. However, its levels are hardly rate-limiting. This fact is also pertinent to developing ATX inhibitors. Those ATX substrate analog inhibitors are difficult to work in vivo, due to the competitive high concentrations of LPC present in the human blood and/or other tissues.

### 2.3. Lysophosphatidylinositol (LPI) and Other LPLs

While phosphatidylinositol (PI) is the substrate of PI3K, one of the most pertinent signaling pathways in cancer [138], LPI as a signaling molecule is much less studied. We have shown that the plasma and ascites levels of LPI in EOC are elevated. In healthy controls, the plasma levels of LPI are in the range of 0–1.5 µM in healthy subjects, which are increased to 1.1–3.0 µM in patients with EOC [28,35]. The means and SDs levels of LPI in non-malignant ascites vs. malignant EOC are 2.9 ± 2.0 µM and 14.7 ± 9.7 µM respectively (Table 1). However, in our lab, unlike LPA and sphingosine-1-phosphate (S1P), neither positive nor negative effects of LPI have been detected in EOC cells. A very recent report has shown that LPC and LPI regulate gene expression, including adhesion molecules, cytokines, and chemokines, as well as those involved in cholesterol biosynthesis (by LPC), or gene transcripts critical for the metabolism of glucose, lipids, and amino acids (by LPI) in human aortic endothelial cells (HAECs). Moreover, LPC and LPI share the ability to transdifferentiate HAECs into innate immune cells [139].

Although a specific receptor of LPI has been reported [140], they warrant further validation for their rules and signaling in cancers. We and others also detected several other LPLs in EOC plasma and/or ascites, including lysophosphatidylethanolamine (LPE), lysophosphatidylglycerol (LPG), lysophosphatidylserine (LPS), lyso-platelet activating factor (lyso-PAF), and PAF [28,35,45,141]. However, the role and signaling of these LPLs in EOC are much less studied.

### 2.4. Sphingosine-1-Phosphate (S1P)

#### 2.4.1. S1P Levels and Production

S1P is the orthologue of LPA with a different backbone (a sphingoid base vs. a glycerol backbone). The physiological and pathologic concentrations of S1P is approximately one order of magnitude lower than that of LPA and is usually present in sub-µM to low µM range [45,46,47,48] (Table 1).

In contrast to LPA, S1P is mainly produced intracellularly by two sphingosine kinases (SphK1 and SphK2; see Figure 1 in [142] and Figure 2 in [143]). S1P may be irreversibly degraded by S1P lyase (SPL) or dephosphorylated by S1P phosphatases (SPPs). Since the S1P lyase level in the blood was much lower than that in tissues and erythrocytes, as well as platelets lack SPL and SPP activity when they mature, higher S1P levels in the blood and lower amounts in tissues are present [144].

Intracellularly produced S1P is exported out of the cell either by the specific transporter Spinster 2 (Spns2) or by several members of the ABC transporter family [145]. This autocrine and/or paracrine action of S1P is known as “inside-out signaling”. In the last few years, it has become evident that S1P also exerts intracellular functions by targeting different molecules, including the PPARγ family factors [145].

#### 2.4.2. S1P Functions and Signaling Mechanisms in EOC

Over the past two decades, increasing evidence demonstrates a strong link between S1P and both normal physiology and progression of different diseases, including cancer and inflammation. S1P may affect survival, proliferation, angiogenesis, and metastatic spread of cancer [144,146].

**Figure 2:**
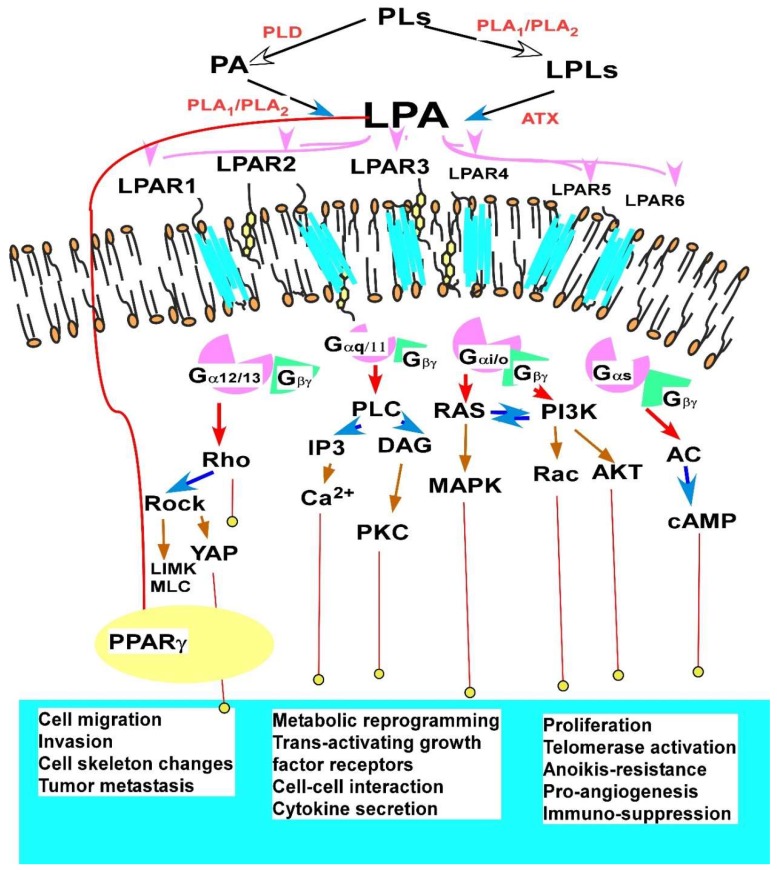
Diagram illustration of LPA receptors, signaling pathways and functions shown in EOC. LPA stimulates almost every aspect of tumor promoting activities [24,25,29,30,31,44,45,70,77,78,79,80,81,82,83,84,85,86,87,88,89,90,91,92,93,94]. This figure illuminates updated information related to LPA receptors (LPAR_1_ to LPAR_6_, and PPARγ), signaling pathways, and functions shown in EOC, modified from a previous review article by Yung et al. [94]. In particular, the nuclear receptor for LPA, PPARγ is included. While Gα12/13, Gαq, and Gαi mediate tumor promoting activities in most cases, Gαs is likely to be a negative regulator counter-reacting Gαi actions. Abbreviations: autotaxin (ATX); LIM kinase (LIMK); lysophospholipids (LPLs); myosin light chain (MLC) phosphatidic acid (PA); phospholipids (PLs); phospholipase D (PLD); phospholipase A_1_ (PLA_1_); and phospholipase A_2_ (PLA_2_).

LPA and S1P share structural similarity. In addition, LPAR_1–3_ and S1PR_1–5_ belong to the same edg-receptor family [147]. Moreover, S1P has been shown to have many similar tumor promoting activities as LPA, and is considered as a cancer treatment target [148,149], which has been reviewed extensively [32,144,146,150,151] (Figure 2). However, there are several major differences between LPA and S1P. Most of all, while LPA displays, in most cases, tumor promoting activities; S1P is multi-facet at several levels, which is emphasized as follows.

Firstly, S1P has strong concentration dependent differential effects. As mentioned above, the physiological/pathological concentrations of S1P are in general lower than those of LPA [46,47,48]. The effects of S1P in EOC cells tested are highly concentration-dependent [152,153,154,155,156,157]. While lower concentrations of S1P (≤1 µM) are usually stimulatory, higher concentrations (10–30 µM) of S1P are inhibitory. The S1P effects are also dependent on cell culture conditions. For example, S1P (10 µM) induced cell death when cells were in suspension but stimulated cell growth when cells were attached. The calcium-dependent induction of cell death by S1P is apparently associated with its inhibitory effect on cell attachment and cell adhesion [152]. N-cadherin, γ- and β-catenins, FAK, and integrin β1 are among the proteins affected by S1P and/or LPA [152,156].

Based on chemically measured S1P concentrations in biological fluids and the binding affinities of S1P to its receptors (in nM to low µM range [158]), the effects of low concentrations of S1P (≤1 μM) may be more pathophysiologically relevant. The effects of high concentrations of S1P (10–30 μM) may be more artificial and/or non-specific.

Secondly, SphK1 and SphK2 have distinct cellular locations, regulations and functions. In general, SphK1 is tumor promoting and SphK2 is suppressive; SphK1 is upregulated in cancer, while SphK2 is downregulated [151,159,160]. Numerous tumor promoting agonists including TNF-α and other inflammatory signaling molecules, such as IL-1β, IFN-γ, IgE, and C5a, stimulate cytosolic SphK1, which translocates to the plasma membrane and uses sphingosine as a substrate to generate S1P. Elevated SphK1 has been shown in EOC cells and functionally involved in drug-resistance and other tumor promoting activities [161,162]. In contrast, SphK2 is located in cytosol or in the nucleus [144]. S1P produced by SphK2 inhibits histone deacetylases (HDACs), which modulates the dynamic balance of histone acetylation and influences the epigenetic regulation of specific target genes [163]. The two SphKs are also likely to have cooperative roles as evidence by knockout mice. Double-knockout animals were embryonic lethal, due to the incomplete maturation of the vascular system and brain, although mice deficient in either SphK1 or SphK2 had no obvious abnormalities [151].

Thirdly, different and opposing effects are mediated by different S1PRs. S1P receptors have been identified so far and named S1PR_1–5_ (formerly referred to as endothelial differentiation gene (Edg1, 5, 3, 6, 8) [147]. Following receptor activation, multiple signaling cascades are activated, which are very similar to or opposing to those stimulated by LPA [164,165]. Among the five S1PRs, S1PR_1_/S1PR_3_ and S1PR_2_ receptors may mediate opposing effects [149,151,153,154,157,159,160]. S1PR_1_ and S1PR_3_ mediate S1P’s tumor promoting activities, such as cell migration and invasion via activation of Rac. Blockage of SphK1, but not SphK2, or S1PR_1/3_ could attenuate ovarian cancer angiogenesis and inhibit angiogenic factor expression in a mouse model [159]. S1PR_1_ is upregulated in ovarian cancer tissues and cell lines, which is negatively regulated by miR-148a in EOC cells [166]. On the other hand, S1PR_2_ generally mediates the inhibitory effect via Rho-mediated inhibition of Rac [160]. S1PR_2_ is also involved in negative regulation of tumor angiogenesis and tumor growth in vivo via RhoC activation [167], although one study has shown that the growth of SKOV3 cells could be decreased by S1PR_2_ inhibition in vitro and in vivo [168]. In addition, S1PR_2_ has an inhibitor role in macrophage recruitment during inflammation [169]. Goetzl et al. reported that both S1PR_2_ and S1PR_3_ are expressed higher in ovarian surface epithelial cells than in ovarian cancer cells [170].

Finally, S1P may have profound regulator effects on inflammation and in the immune system. The SphKs/S1P/S1PR_1_ axis plays an important role in the immune regulation. It is involved in the mature vascular system; pathological angiogenesis; immune cell egress from tissue compartments; hematopoietic, vascular, and stem cell survival; and cytokine production. In particular, S1P induces STAT3 activation in tumor-associated myeloid derived suppressing cells (MDSCs) [151]. In addition, the roles of LPA and S1P on angiogenesis are likely to be different. S1P may have a direct proangiogenic role on ECs [159]. S1P and its receptors are involved in vessel morphogenesis and angiogenesis during embryonic development and in the adult organism both under normal and pathological conditions [171,172]. On the other hand, LPA’s role on ECs may be indirect and mediated by its effect on tumor and/or TME cells via releasing proangiogenic factors, such as IL-8 [92,94,173].

SphK1 is highly expressed in the tumor stroma of high grade serous ovarian cancer (HGSOC) and is required for the differentiation and tumor promoting function of cancer-associated fibroblasts (CAFs) [174]. While increasing S1P catabolism or inhibiting S1P biosynthesis could become a new way to treat cancer, some studies found that the inhibition of S1P raised secondary malignancy [151,175].

A biospecific monoclonal antibody to S1P (S1P mAb) has been developed and investigated for its role in tumorigenesis. The anti-S1P mAb substantially reduced tumor progression and in some cases eliminated measurable tumors in murine xenograft and allograft models. Tumor growth inhibition was attributed to antiangiogenic and antitumorigenic effects of the antibody [176]. The anti-S1P mAb blocked EC migration and resulting capillary formation, inhibited blood vessel formation induced by VEGF and bFGF, and arrested tumor-associated angiogenesis [176]. In this study, SKOV3 cells were used for ovarian cancer, but they are not cells from HGSOC, which accounts for about 70% of EOC cases, with less than 30% of patients currently surviving more than five years after diagnosis with little improvement in overall survival over the past 40 years [177,178,179]. Hence, the therapeutic significance of targeting S1P in EOC warrants further studies.

In summary, the role of S1P in the pathogenesis of ovarian cancer remains unclear and controversial and more studies are clearly required. Due to the multi-faceted nature of S1P’s roles and signaling, targeting S1P signaling may be a double-edged sword.

### 2.5. Sphingosylphosphorylcholine (SPC)

SPC is an orthologue of LPC with a different backbone (a sphingoid base vs. a glycerol backbone). The levels of SPC in EOC vs. non-malignant ascites is low: 71.5 ± 50.8 nM vs. 17.9 ± 10.1 nM, respectively [28]. The levels of plasma SPC are also at nM range [45]. SPC is a potential calcium-release inducer in EOC cells [25,26,29]. SPC also shows other cellular activities in EOC cells, including regulation of IL-8 expression in EOC cells [94]. However, high concentrations (at µM level) of SPC is very toxic to cells. SPC induces dendritic cells (DC) chemotaxis and stimulates the production of IL-12 from DC [180,181]. However, the real physiological or pathological roles of SPC in EOC are still very elusive.

## 3. Conclusions

The reciprocal interplay of cancer cells and TME is an indispensable prerequisite for tumor growth and progression. Ovarian cancer, the most lethal of all gynecological malignancies, is characterized by a unique TME that enables specific and efficient metastatic mechanisms/routes, impairs immune surveillance, and mediates therapy resistance. More specifically, detached cancer cells—as well as large numbers of T cells, TAMs, and other host cells—cooperate with resident host cells to support tumor progression and immune evasion. The presence of the peritoneal fluid (ascites) enables more efficient tumor-stromal cell interactions and the transcoelomic spread of tumor cells to other pelvic and peritoneal organs. In particular, this fluid is rich in tumor-promoting soluble factors including elevated LPLs, either in EV or non-EV forms. Several important future directions and unresolved questions include, but are not limited to: development of standard and uniform methods for lipid extraction and analyses; further characterization of LPL regulation (both production and degradation) and their signaling mechanisms; development of strategies for cancer-specific targeting those tumor promoting lipids; and conducting more studies on their extracellular associations in order to better develop markers and targeting. Overall, it is critical to take TME into consideration to develop the next generation of therapeutic strategies.

## Figures and Tables

**Table 1:** Concentrations (in µM) of major lysophospholipids (LPLs) involved in epithelial ovarian cancer (EOC).

Lipid	EOC or BC Plasma	Healthy Control Plasma	EOC Ascites	Benign Ascites
Acyl-LPA	2–22 [33,34]	0.6–0.9 [33,34]	19–95 [28,43]	2.9 [28,43]
Alkyl-, and alkenyl-LPA			3.7 ± 1.7 [28]	0.9 ± 0.7 [28]
LPI	to 3.0 [28,35]	0–1.5 [28,35]	14.7 ± 9.7 [28,35]	2.9 ± 2.0 [28,35]
LPC	120 ± 0.30 [45] 117–153 [34]	128 ± 46 [45] 122 [34]		
S1P	0.52 ± 0.12 [137]	0.58 ± 0.18 [45,46,47,48]	sub-µM to low µM [45,46,47,48]	sub-µM to low µM [45,46,47,48]

BC: breast cancer.

## References

[1] Westermann A.M., Beijnen J.H., Moolenaar W.H., Rodenhuis S. (1997). Growth factors in human ovarian cancer. Cancer Treat. Rev..

[2] Amsterdam A. (2010). Novel role of growth factors in ovary function. Harefuah.

[3] Thibault B., Castells M., Delord J.P., Couderc B. (2014). Ovarian cancer microenvironment: Implications for cancer dissemination and chemoresistance acquisition. Cancer Metastasis Rev..

[4] Luo Z., Wang Q., Lau W.B., Lau B., Xu L., Zhao L., Yang H., Feng M., Xuan Y., Yang Y. (2016). Tumor microenvironment: The culprit for ovarian cancer metastasis?. Cancer Lett..

[5] Hodeib M., Serna-Gallegos T., Tewari K.S. (2015). A review of HER2-targeted therapy in breast and ovarian cancer: Lessons from antiquity—Cleopatra and Penelope. Future Oncol..

[6] Ntanasis-Stathopoulos I., Fotopoulos G., Tzanninis I.G., Kotteas E.A. (2016). The emerging role of tyrosine kinase inhibitors in ovarian cancer treatment: A systematic review. Cancer Investig..

[7] Cai H., Chiorean E.G., Chiorean M.V., Rex D.K., Robb B.W., Hahn N.M., Liu Z., Loehrer P.J., Harrison M.L., Xu Y. (2013). Elevated phospholipase A2 activities in plasma samples from multiple cancers. PLoS ONE.

[8] Pap E., Pallinger E., Pasztoi M., Falus A. (2009). Highlights of a new type of intercellular communication: Microvesicle-based information transfer. Inflamm. Res..

[9] He C., Zheng S., Luo Y., Wang B. (2018). Exosome theranostics: Biology and translational medicine. Theranostics.

[10] Taylor D.D., Gercel-Taylor C. (2008). MicroRNA signatures of tumor-derived exosomes as diagnostic biomarkers of ovarian cancer. Gynecol. Oncol..

[11] Beach A., Zhang H.G., Ratajczak M.Z., Kakar S.S. (2014). Exosomes: An overview of biogenesis, composition and role in ovarian cancer. J. Ovarian Res..

[12] Saleem S.N., Abdel-Mageed A.B. (2015). Tumor-derived exosomes in oncogenic reprogramming and cancer progression. Cell. Mol. Life Sci..

[13] Cheng L., Wu S., Zhang K., Qing Y., Xu T. (2017). A comprehensive overview of exosomes in ovarian cancer: Emerging biomarkers and therapeutic strategies. J. Ovarian Res..

[14] Hanahan D., Weinberg R.A. (2011). Hallmarks of cancer: The next generation. Cell.

[15] Amoroso M.R., Matassa D.S., Agliarulo I., Avolio R., Maddalena F., Condelli V., Landriscina M., Esposito F. (2017). Stress-adaptive response in ovarian cancer drug resistance: Role of trap1 in oxidative metabolism-driven inflammation. Adv. Protein Chem. Struct. Biol..

[16] Ke C., Li A., Hou Y., Sun M., Yang K., Cheng J., Wang J., Ge T., Zhang F., Li Q. (2016). Metabolic phenotyping for monitoring ovarian cancer patients. Sci. Rep..

[17] Fahy E., Subramaniam S., Murphy R.C., Nishijima M., Raetz C.R., Shimizu T., Spener F., van Meer G., Wakelam M.J., Dennis E.A. (2009). Update of the lipid maps comprehensive classification system for lipids. J. Lipid Res..

[18] Tania M., Khan M.A., Song Y. (2010). Association of lipid metabolism with ovarian cancer. Curr. Oncol..

[19] Tsujiuchi T., Araki M., Hirane M., Dong Y., Fukushima N. (2014). Lysophosphatidic acid receptors in cancer pathobiology. Histol. Histopathol..

[20] Pua T.L., Wang F.Q., Fishman D.A. (2009). Roles of LPA in ovarian cancer development and progression. Future Oncol..

[21] Tokumura A. (2002). Physiological and pathophysiological roles of lysophosphatidic acids produced by secretory lysophospholipase d in body fluids. Biochim. Biophys. Acta.

[22] Turkoglu O., Zeb A., Graham S., Szyperski T., Szender J.B., Odunsi K., Bahado-Singh R. (2016). Metabolomics of biomarker discovery in ovarian cancer: A systematic review of the current literature. Metabolomics.

[23] Varas-Godoy M., Rice G., Illanes S.E. (2017). The crosstalk between ovarian cancer stem cell niche and the tumor microenvironment. Stem Cells Int..

[24] Ray U., Roy Chowdhury S., Vasudevan M., Bankar K., Roychoudhury S., Roy S.S. (2017). Gene regulatory networking reveals the molecular cue to lysophosphatidic acid-induced metabolic adaptations in ovarian cancer cells. Mol. Oncol..

[25] Xu Y., Fang X.J., Casey G., Mills G.B. (1995). Lysophospholipids activate ovarian and breast cancer cells. Biochem. J..

[26] Xu Y., Gaudette D.C., Boynton J.D., Frankel A., Fang X.J., Sharma A., Hurteau J., Casey G., Goodbody A., Mellors A. (1995). Characterization of an ovarian cancer activating factor in ascites from ovarian cancer patients. Clin. Cancer Res..

[27] Benesch M.G.K., MacIntyre I.T.K., McMullen T.P.W., Brindley D.N. (2018). Coming of age for autotaxin and lysophosphatidate signaling: Clinical applications for preventing, detecting and targeting tumor-promoting inflammation. Cancers.

[28] Xiao Y.J., Schwartz B., Washington M., Kennedy A., Webster K., Belinson J., Xu Y. (2001). Electrospray ionization mass spectrometry analysis of lysophospholipids in human ascitic fluids: Comparison of the lysophospholipid contents in malignant vs. nonmalignant ascitic fluids. Anal. Biochem..

[29] Xu Y., Casey G., Mills G.B. (1995). Effect of lysophospholipids on signaling in the human jurkat T cell line. J. Cell. Physiol..

[30] Sengupta S., Wang Z., Tipps R., Xu Y. (2004). Biology of lpa in health and disease. Semin. Cell Dev. Biol..

[31] Mills G.B., Moolenaar W.H. (2003). The emerging role of lysophosphatidic acid in cancer. Nat. Rev. Cancer.

[32] Murph M., Mills G.B. (2007). Targeting the lipids LPA and S1p and their signalling pathways to inhibit tumour progression. Expert. Rev. Mol. Med..

[33] Xu Y., Shen Z., Wiper D.W., Wu M., Morton R.E., Elson P., Kennedy A.W., Belinson J., Markman M., Casey G. (1998). Lysophosphatidic acid as a potential biomarker for ovarian and other gynecologic cancers. JAMA.

[34] Sutphen R., Xu Y., Wilbanks G.D., Fiorica J., Grendys E.C., LaPolla J.P., Arango H., Hoffman M.S., Martino M., Wakeley K. (2004). Lysophospholipids are potential biomarkers of ovarian cancer. Cancer Epidemiol. Biomarkers Prev..

[35] Xiao Y., Chen Y., Kennedy A.W., Belinson J., Xu Y. (2000). Evaluation of plasma lysophospholipids for diagnostic significance using electrospray ionization mass spectrometry (ESI-MS) analyses. Ann. N. Y. Acad. Sci..

[36] Sedlakova I., Vavrova J., Tosner J., Hanousek L. (2006). Lysophosphatidic acid in ovarian cancer patients. Ceska Gynekol..

[37] Meleh M., Pozlep B., Mlakar A., Meden-Vrtovec H., Zupancic-Kralj L. (2007). Determination of serum lysophosphatidic acid as a potential biomarker for ovarian cancer. J. Chromatogr. B Analyt. Technol. Biomed. Life Sci..

[38] Sedlakova I., Vavrova J., Tosner J., Hanousek L. (2008). Lysophosphatidic acid: An ovarian cancer marker. Eur. J. Gynaecol. Oncol..

[39] Nakamura K., Igarashi K., Ohkawa R., Yokota H., Masuda A., Nakagawa S., Yano T., Ikeda H., Aoki J., Yatomi Y. (2012). Serum autotaxin is not a useful biomarker for ovarian cancer. Lipids.

[40] Lu Z., Chen Y., Hu Z., Hu C. (2015). Diagnostic value of total plasma lysophosphatidic acid in ovarian cancer: A meta-analysis. Int. J. Gynecol. Cancer.

[41] Zhang Y.J., Cao L.Y., Fu Z.Z., Wang Y.J., Wang G.X., Gu T. (2015). Clinical significance of plasma lysophosphatidic acid levels in the differential diagnosis of ovarian cancer. J. Cancer Res. Ther..

[42] Li Y.Y., Zhang W.C., Zhang J.L., Zheng C.J., Zhu H., Yu H.M., Fan L.M. (2015). Plasma levels of lysophosphatidic acid in ovarian cancer versus controls: A meta-analysis. Lipids Health Dis..

[43] Westermann A.M., Havik E., Postma F.R., Beijnen J.H., Dalesio O., Moolenaar W.H., Rodenhuis S. (1998). Malignant effusions contain lysophosphatidic acid (LPA)-like activity. Ann. Oncol..

[44] Cai Q., Zhao Z., Antalis C., Yan L., Del Priore G., Hamed A.H., Stehman F.B., Schilder J.M., Xu Y. (2012). Elevated and secreted phospholipase A(2) activities as new potential therapeutic targets in human epithelial ovarian cancer. FASEB J..

[45] Murph M., Tanaka T., Pang J., Felix E., Liu S., Trost R., Godwin A.K., Newman R., Mills G. (2007). Liquid chromatography mass spectrometry for quantifying plasma lysophospholipids: Potential biomarkers for cancer diagnosis. Methods Enzymol..

[46] Zhao Z., Xu Y. (2010). An extremely simple method for extraction of lysophospholipids and phospholipids from blood samples. J. Lipid Res..

[47] Michels M., Japtok L., Alisjahbana B., Wisaksana R., Sumardi U., Puspita M., Kleuser B., de Mast Q., van der Ven A.J. (2015). Decreased plasma levels of the endothelial protective sphingosine-1-phosphate are associated with dengue-induced plasma leakage. J. Infect..

[48] Ramanathan R., Raza A., Sturgill J., Lyon D., Young J., Hait N.C., Takabe K. (2017). Paradoxical association of postoperative plasma sphingosine-1-phosphate with breast cancer aggressiveness and chemotherapy. Mediators Inflamm..

[49] Ma L., Uchida H., Nagai J., Inoue M., Aoki J., Ueda H. (2010). Evidence for de novo synthesis of lysophosphatidic acid in the spinal cord through phospholipase A2 and autotaxin in nerve injury-induced neuropathic pain. J. Pharmacol. Exp. Ther..

[50] Eder A.M., Sasagawa T., Mao M., Aoki J., Mills G.B. (2000). Constitutive and lysophosphatidic acid (LPA)-induced LPA production: Role of phospholipase D and phospholipase a2. Clin. Cancer Res..

[51] Sengupta S., Xiao Y.J., Xu Y. (2003). A novel laminin-induced LPA autocrine loop in the migration of ovarian cancer cells. FASEB J..

[52] Shen Z., Belinson J., Morton R.E., Xu Y., Xu Y. (1998). Phorbol 12-myristate 13-acetate stimulates lysophosphatidic acid secretion from ovarian and cervical cancer cells but not from breast or leukemia cells. Gynecol. Oncol..

[53] Aoki J. (2004). Mechanisms of lysophosphatidic acid production. Semin. Cell. Dev. Biol..

[54] Benesch M.G., Tang X., Venkatraman G., Bekele R.T., Brindley D.N. (2016). Recent advances in targeting the autotaxin-lysophosphatidate-lipid phosphate phosphatase axis in vivo. J. Biomed. Res..

[55] Nikolaou A., Kokotou M.G., Limnios D., Psarra A., Kokotos G. (2017). Autotaxin inhibitors: A patent review (2012–2016). Expert. Opin. Ther. Pat..

[56] Gaits F., Fourcade O., Le Balle F., Gueguen G., Gaige B., Gassama-Diagne A., Fauvel J., Salles J.P., Mauco G., Simon M.F. (1997). Lysophosphatidic acid as a phospholipid mediator: Pathways of synthesis. FEBS Lett..

[57] Pages C., Simon M.F., Valet P., Saulnier-Blache J.S. (2001). Lysophosphatidic acid synthesis and release. Prostaglandins Other Lipid Mediat..

[58] Roszkowski I., Niewiarowska M., Czerwinska J., Bar-Pratkowska J., Obrebski T. (1971). Problems of surgical treatment of a patient with blood platelet disorders. Ginekol. Pol..

[59] Hisada Y., Geddings J.E., Ay C., Mackman N. (2015). Venous thrombosis and cancer: From mouse models to clinical trials. J. Thromb. Haemost..

[60] Menczer J. (2017). Preoperative elevated platelet count and thrombocytosis in gynecologic malignancies. Arch. Gynecol. Obstet..

[61] Swier N., Versteeg H.H. (2017). Reciprocal links between venous thromboembolism, coagulation factors and ovarian cancer progression. Thromb. Res..

[62] Zhou Q., Huang F., He Z., Zuo M.Z. (2018). Clinicopathological and prognostic significance of platelet count in patients with ovarian cancer. Climacteric.

[63] Lin R.J., Afshar-Kharghan V., Schafer A.I. (2014). Paraneoplastic thrombocytosis: The secrets of tumor self-promotion. Blood.

[64] Nugent D., Belinson J.L., Xu Y. (1999). The synergistic interactions of oleoyl-lysophosphatidic acid in platelet aggregation. Med. Sci. Res..

[65] Nugent D., Xu Y. (2000). Sphingosine-1-phosphate: Characterization of its inhibition of platelet aggregation. Platelets.

[66] Leblanc R., Houssin A., Peyruchaud O. (2018). Platelets, autotaxin and lysophosphatidic acid signaling: Win-win factors for cancer metastasis. Br. J. Pharmacol..

[67] Krishnan V., Clark R., Chekmareva M., Johnson A., George S., Shaw P., Seewaldt V., Rinker-Schaeffer C. (2015). In vivo and ex vivo approaches to study ovarian cancer metastatic colonization of milky spot structures in peritoneal adipose. J. Vis. Exp..

[68] Feist P.E., Loughran E.A., Stack M.S., Hummon A.B. (2018). Quantitative proteomic analysis of murine white adipose tissue for peritoneal cancer metastasis. Anal. Bioanal. Chem..

[69] Cai Q., Yan L., Xu Y. (2015). Anoikis resistance is a critical feature of highly aggressive ovarian cancer cells. Oncogene.

[70] Nieman K.M., Kenny H.A., Penicka C.V., Ladanyi A., Buell-Gutbrod R., Zillhardt M.R., Romero I.L., Carey M.S., Mills G.B., Hotamisligil G.S. (2011). Adipocytes promote ovarian cancer metastasis and provide energy for rapid tumor growth. Nat. Med..

[71] Umezu-Goto M., Kishi Y., Taira A., Hama K., Dohmae N., Takio K., Yamori T., Mills G.B., Inoue K., Aoki J. (2002). Autotaxin has lysophospholipase d activity leading to tumor cell growth and motility by lysophosphatidic acid production. J. Cell. Biol..

[72] Benesch M.G., Zhao Y.Y., Curtis J.M., McMullen T.P., Brindley D.N. (2015). Regulation of autotaxin expression and secretion by lysophosphatidate and sphingosine 1-phosphate. J. Lipid Res..

[73] Volden P.A., Skor M.N., Johnson M.B., Singh P., Patel F.N., McClintock M.K., Brady M.J., Conzen S.D. (2016). Mammary adipose tissue-derived lysophospholipids promote estrogen receptor-negative mammary epithelial cell proliferation. Cancer Prev. Res..

[74] Dusaulcy R., Rancoule C., Gres S., Wanecq E., Colom A., Guigne C., van Meeteren L.A., Moolenaar W.H., Valet P., Saulnier-Blache J.S. (2011). Adipose-specific disruption of autotaxin enhances nutritional fattening and reduces plasma lysophosphatidic acid. J. Lipid Res..

[75] Benesch M.G.K., Yang Z., Tang X., Meng G., Brindley D.N. (2017). Lysophosphatidate signaling: The tumor microenvironment’s new nemesis. Trends Cancer.

[76] Ren J., Xiao Y.J., Singh L.S., Zhao X., Zhao Z., Feng L., Rose T.M., Prestwich G.D., Xu Y. (2006). Lysophosphatidic acid is constitutively produced by human peritoneal mesothelial cells and enhances adhesion, migration, and invasion of ovarian cancer cells. Cancer Res..

[77] Knowlden S., Georas S.N. (2014). The autotaxin-LPA axis emerges as a novel regulator of lymphocyte homing and inflammation. J. Immunol..

[78] Halama A., Guerrouahen B.S., Pasquier J., Satheesh N.J., Suhre K., Rafii A. (2017). Nesting of colon and ovarian cancer cells in the endothelial niche is associated with alterations in glycan and lipid metabolism. Sci. Rep..

[79] Wong J.L., Obermajer N., Odunsi K., Edwards R.P., Kalinski P. (2016). Synergistic COX2 induction by IFNgamma and TNFalpha self-limits type-1 immunity in the human tumor microenvironment. Cancer Immunol. Res..

[80] Reinartz S., Finkernagel F., Adhikary T., Rohnalter V., Schumann T., Schober Y., Nockher W.A., Nist A., Stiewe T., Jansen J.M. (2016). A transcriptome-based global map of signaling pathways in the ovarian cancer microenvironment associated with clinical outcome. Genome Biol..

[81] Nakayama J., Raines T.A., Lynch K.R., Slack-Davis J.K. (2015). Decreased peritoneal ovarian cancer growth in mice lacking expression of lipid phosphate phosphohydrolase 1. PLoS ONE.

[82] Baudhuin L.M., Cristina K.L., Lu J., Xu Y. (2002). AKT activation induced by lysophosphatidic acid and sphingosine-1-phosphate requires both mitogen-activated protein kinase kinase and p38 mitogen-activated protein kinase and is cell-line specific. Mol. Pharmacol..

[83] Cai H., Xu Y. (2013). The role of lpa and yap signaling in long-term migration of human ovarian cancer cells. Cell Commun. Signal..

[84] Fan Q., Cai Q., Li P., Wang W., Wang J., Gerry E., Wang T.L., Shih I.M., Nephew K.P., Xu Y. (2017). The novel zip4 regulation and its role in ovarian cancer. Oncotarget.

[85] Fan Q., Cai Q., Xu Y. (2017). LPA Regulates sox9 in Ovarian Cancer Cells.

[86] Fang X., Yu S., Bast R.C., Liu S., Xu H.J., Hu S.X., LaPushin R., Claret F.X., Aggarwal B.B., Lu Y. (2004). Mechanisms for lysophosphatidic acid-induced cytokine production in ovarian cancer cells. J. Biol. Chem..

[87] Ha J.H., Ward J.D., Radhakrishnan R., Jayaraman M., Song Y.S., Dhanasekaran D.N. (2016). Lysophosphatidic acid stimulates epithelial to mesenchymal transition marker slug/snail2 in ovarian cancer cells via galphai2, src, and hif1alpha signaling nexus. Oncotarget.

[88] Jiang Y., Berk M., Singh L.S., Tan H., Yin L., Powell C.T., Xu Y. (2005). Kiss1 suppresses metastasis in human ovarian cancer via inhibition of protein kinase c alpha. Clin. Exp. Metastasis.

[89] Kim K.S., Sengupta S., Berk M., Kwak Y.G., Escobar P.F., Belinson J., Mok S.C., Xu Y. (2006). Hypoxia enhances lysophosphatidic acid responsiveness in ovarian cancer cells and lysophosphatidic acid induces ovarian tumor metastasis in vivo. Cancer Res..

[90] Li H., Wang D., Zhang H., Kirmani K., Zhao Z., Steinmetz R., Xu Y. (2009). Lysophosphatidic acid stimulates cell migration, invasion, and colony formation as well as tumorigenesis/metastasis of mouse ovarian cancer in immunocompetent mice. Mol. Cancer Ther..

[91] Li H., Zhao Z., Wei G., Yan L., Wang D., Zhang H., Sandusky G.E., Turk J., Xu Y. (2010). Group via phospholipase a2 in both host and tumor cells is involved in ovarian cancer development. FASEB J..

[92] Lu J., Xiao Yj Y.J., Baudhuin L.M., Hong G., Xu Y. (2002). Role of ether-linked lysophosphatidic acids in ovarian cancer cells. J. Lipid Res..

[93] Ren J., Li Y., Zhang Y.L., Zhou X.H., Zhang L., Yang Y., Li Y. (2010). Effect of inhibitors of phospholipase A(2); on the metastasis potentials of human ovarian cancer cells. Xi Bao Yu Fen Zi Mian Yi Xue Za Zhi.

[94] Schwartz B.M., Hong G., Morrison B.H., Wu W., Baudhuin L.M., Xiao Y.J., Mok S.C., Xu Y. (2001). Lysophospholipids increase interleukin-8 expression in ovarian cancer cells. Gynecol. Oncol..

[95] Sengupta S., Kim K.S., Berk M.P., Oates R., Escobar P., Belinson J., Li W., Lindner D.J., Williams B., Xu Y. (2007). Lysophosphatidic acid downregulates tissue inhibitor of metalloproteinases, which are negatively involved in lysophosphatidic acid-induced cell invasion. Oncogene.

[96] Fang X., Gaudette D., Furui T., Mao M., Estrella V., Eder A., Pustilnik T., Sasagawa T., Lapushin R., Yu S. (2000). Lysophospholipid growth factors in the initiation, progression, metastases, and management of ovarian cancer. Ann. N. Y. Acad. Sci..

[97] Fang X., Schummer M., Mao M., Yu S., Tabassam F.H., Swaby R., Hasegawa Y., Tanyi J.L., LaPushin R., Eder A. (2002). Lysophosphatidic acid is a bioactive mediator in ovarian cancer. Biochim. Biophys. Acta.

[98] Mills G.B., Eder A., Fang X., Hasegawa Y., Mao M., Lu Y., Tanyi J., Tabassam F.H., Wiener J., Lapushin R. (2002). Critical role of lysophospholipids in the pathophysiology, diagnosis, and management of ovarian cancer. Cancer Treat. Res..

[99] Yung Y.C., Stoddard N.C., Chun J. (2014). Lpa receptor signaling: Pharmacology, physiology, and pathophysiology. J. Lipid Res..

[100] Jesionowska A., Cecerska-Heryc E., Matoszka N., Dolegowska B. (2015). Lysophosphatidic acid signaling in ovarian cancer. J. Recept Signal. Transduct. Res..

[101] Wang G.L., Wen Z.Q., Xu W.P., Wang Z.Y., Du X.L., Wang F. (2008). Inhibition of lysophosphatidic acid receptor-2 expression by rna interference decreases lysophosphatidic acid-induced urokinase plasminogen activator activation, cell invasion, and migration in ovarian cancer SKOV-3 cells. Croat Med. J..

[102] Yang K., Zheng D., Deng X., Bai L., Xu Y., Cong Y.S. (2008). Lysophosphatidic acid activates telomerase in ovarian cancer cells through hypoxia-inducible factor-1alpha and the PI3K pathway. J. Cell Biochem..

[103] Bai C.Q., Yao Y.W., Liu C.H., Zhang H., Xu X.B., Zeng J.L., Liang W.J., Yang W., Song Y. (2014). Diagnostic and prognostic significance of lysophosphatidic acid in malignant pleural effusions. J. Thorac. Dis..

[104] Fan Q., Cai Q., Xu Y. (2015). Foxm1 is a downstream target of lpa and yap oncogenic signaling pathways in high grade serous ovarian cancer. Oncotarget.

[105] Fishman D.A., Liu Y., Ellerbroek S.M., Stack M.S. (2001). Lysophosphatidic acid promotes matrix metalloproteinase (MMP) activation and mmp-dependent invasion in ovarian cancer cells. Cancer Res..

[106] Burkhalter R.J., Westfall S.D., Liu Y., Stack M.S. (2015). Lysophosphatidic acid initiates epithelial to mesenchymal transition and induces beta-catenin-mediated transcription in epithelial ovarian carcinoma. J. Biol. Chem..

[107] Cui J., Liu Y., Wang X. (2017). The roles of glycodelin in cancer development and progression. Front. Immunol..

[108] Xu Y., Xiao Y.J., Zhu K., Baudhuin L.M., Lu J., Hong G., Kim K.S., Cristina K.L., Song L., F S.W. (2003). Unfolding the pathophysiological role of bioactive lysophospholipids. Curr. Drug Targets Immune Endocr. Metabol. Disord..

[109] Li H., Xu Y. (2007).

[110] Seo E.J., Kwon Y.W., Jang I.H., Kim D.K., Lee S.I., Choi E.J., Kim K.H., Suh D.S., Lee J.H., Choi K.U. (2016). Autotaxin regulates maintenance of ovarian cancer stem cells through lysophosphatidic acid-mediated autocrine mechanism. Stem Cells.

[111] Bar-Shavit R., Maoz M., Kancharla A., Nag J.K., Agranovich D., Grisaru-Granovsky S., Uziely B. (2016). G protein-coupled receptors in cancer. Int. J. Mol. Sci..

[112] Taniguchi R., Inoue A., Sayama M., Uwamizu A., Yamashita K., Hirata K., Yoshida M., Tanaka Y., Kato H.E., Nakada-Nakura Y. (2017). Structural insights into ligand recognition by the lysophosphatidic acid receptor LPA6. Nature.

[113] Hope J.M., Wang F.Q., Whyte J.S., Ariztia E.V., Abdalla W., Long K., Fishman D.A. (2009). LPA receptor 2 mediates LPA-induced endometrial cancer invasion. Gynecol. Oncol..

[114] Lin S., Wang D., Iyer S., Ghaleb A.M., Shim H., Yang V.W., Chun J., Yun C.C. (2009). The absence of lpa2 attenuates tumor formation in an experimental model of colitis-associated cancer. Gastroenterology.

[115] Stoddard N.C., Chun J. (2015). Promising pharmacological directions in the world of lysophosphatidic acid signaling. Biomol. Ther..

[116] Takahashi K., Fukushima K., Onishi Y., Inui K., Node Y., Fukushima N., Honoki K., Tsujiuchi T. (2017). Lysophosphatidic acid (LPA) signaling via LPA4 and LPA6 negatively regulates cell motile activities of colon cancer cells. Biochem. Biophys. Res. Commun..

[117] Ishii S., Hirane M., Fukushima K., Tomimatsu A., Fukushima N., Tsujiuchi T. (2015). Diverse effects of LPA4, LPA5 and LPA6 on the activation of tumor progression in pancreatic cancer cells. Biochem. Biophys. Res. Commun..

[118] Takahashi K., Fukushima K., Otagaki S., Ishimoto K., Minami K., Fukushima N., Honoki K., Tsujiuchi T. (2018). Effects of LPA1 and LPA6 on the regulation of colony formation activity in colon cancer cells treated with anticancer drugs. J. Recept. Signal. Transduct. Res..

[119] McIntyre T.M., Pontsler A.V., Silva A.R., St Hilaire A., Xu Y., Hinshaw J.C., Zimmerman G.A., Hama K., Aoki J., Arai H. (2003). Identification of an intracellular receptor for lysophosphatidic acid (LPA): LPA is a transcellular ppargamma agonist. Proc. Natl. Acad. Sci. USA.

[120] Tsukahara T., Tsukahara R., Yasuda S., Makarova N., Valentine W.J., Allison P., Yuan H., Baker D.L., Li Z., Bittman R. (2006). Different residues mediate recognition of 1-*O*-oleyllysophosphatidic acid and rosiglitazone in the ligand binding domain of peroxisome proliferator-activated receptor gamma. J. Biol. Chem..

[121] Tsukahara T. (2013). Ppar gamma networks in cell signaling: Update and impact of cyclic phosphatidic acid. J. Lipids.

[122] Tsukahara T., Haniu H., Matsuda Y. (2013). Effect of alkyl glycerophosphate on the activation of peroxisome proliferator-activated receptor gamma and glucose uptake in C2C12 cells. Biochem. Biophys. Res. Commun..

[123] Worzfeld T., Finkernagel F., Reinartz S., Konzer A., Adhikary T., Nist A., Stiewe T., Wagner U., Looso M., Graumann J. (2018). Proteotranscriptomics reveal signaling networks in the ovarian cancer microenvironment. Mol. Cell. Proteom..

[124] Knowlden S.A., Capece T., Popovic M., Chapman T.J., Rezaee F., Kim M., Georas S.N. (2014). Regulation of T cell motility in vitro and in vivo by LPA and LPA2. PLoS ONE.

[125] Okita M., Gaudette D.C., Mills G.B., Holub B.J. (1997). Elevated levels and altered fatty acid composition of plasma lysophosphatidylcholine(lysopc) in ovarian cancer patients. Int. J. Cancer.

[126] Zhang Y., Liu Y., Li L., Wei J., Xiong S., Zhao Z. (2016). High resolution mass spectrometry coupled with multivariate data analysis revealing plasma lipidomic alteration in ovarian cancer in asian women. Talanta.

[127] Zhao Z., Xiao Y., Elson P., Tan H., Plummer S.J., Berk M., Aung P.P., Lavery I.C., Achkar J.P., Li L. (2007). Plasma lysophosphatidylcholine levels: Potential biomarkers for colorectal cancer. J. Clin. Oncol..

[128] Zhao Z., Xu Y. (2009). Measurement of endogenous lysophosphatidic acid by ESI-MS/MS in plasma samples requires pre-separation of lysophosphatidylcholine. J. Chromatogr. B Analyt. Technol. Biomed. Life Sci..

[129] Ross T., Jakubzig B., Grundmann M., Massing U., Kostenis E., Schlesinger M., Bendas G. (2016). The molecular mechanism by which saturated lysophosphatidylcholine attenuates the metastatic capacity of melanoma cells. FEBS Open Bio.

[130] Kuhn T., Floegel A., Sookthai D., Johnson T., Rolle-Kampczyk U., Otto W., von Bergen M., Boeing H., Kaaks R. (2016). Higher plasma levels of lysophosphatidylcholine 18:0 are related to a lower risk of common cancers in a prospective metabolomics study. BMC Med..

[131] Goto T., Terada N., Inoue T., Kobayashi T., Nakayama K., Okada Y., Yoshikawa T., Miyazaki Y., Uegaki M., Utsunomiya N. (2015). Decreased expression of lysophosphatidylcholine (16:0/OH) in high resolution imaging mass spectrometry independently predicts biochemical recurrence after surgical treatment for prostate cancer. Prostate.

[132] Song Y., Wilkins P., Hu W., Murthy K.S., Chen J., Lee Z., Oyesanya R., Wu J., Barbour S.E., Fang X. (2007). Inhibition of calcium-independent phospholipase A2 suppresses proliferation and tumorigenicity of ovarian carcinoma cells. Biochem. J..

[133] Li H., Zhao Z., Antalis C., Zhao Z., Emerson R., Wei G., Zhang S., Zhang Z.Y., Xu Y. (2011). Combination therapy of an inhibitor of group via phospholipase A2 with paclitaxel is highly effective in blocking ovarian cancer development. Am. J. Pathol..

[134] Carneiro A.B., Iaciura B.M., Nohara L.L., Lopes C.D., Veas E.M., Mariano V.S., Bozza P.T., Lopes U.G., Atella G.C., Almeida I.C. (2013). Lysophosphatidylcholine triggers TLR2- and TLR4-mediated signaling pathways but counteracts LPS-induced no synthesis in peritoneal macrophages by inhibiting NF-kappab translocation and MAPK/ERK phosphorylation. PLoS ONE.

[135] Li X., Fang P., Li Y., Kuo Y.M., Andrews A.J., Nanayakkara G., Johnson C., Fu H., Shan H., Du F. (2016). Mitochondrial reactive oxygen species mediate lysophosphatidylcholine-induced endothelial cell activation. Arterioscler. Thromb. Vasc. Biol..

[136] de Bony J., Dufourcq J., Clin B. (1979). Lipid-protein interactions: NMR study of melittin and its binding to lysophosphatidylcholine. Biochim. Biophys. Acta.

[137] Kim Y.L., Im Y.J., Ha N.C., Im D.S. (2007). Albumin inhibits cytotoxic activity of lysophosphatidylcholine by direct binding. Prostaglandins Other Lipid Mediat..

[138] Sanchez-Vega F., Mina M., Armenia J., Chatila W.K., Luna A., La K.C., Dimitriadoy S., Liu D.L., Kantheti H.S., Saghafinia S. (2018). Oncogenic signaling pathways in the cancer genome atlas. Cell.

[139] Li X., Wang L., Fang P., Sun Y., Jiang X., Wang H., Yang X.F. (2018). Lysophospholipids induce innate immune transdifferentiation of endothelial cells, resulting in prolonged endothelial activation. J. Biol. Chem..

[140] Hurst K., Badgley C., Ellsworth T., Bell S., Friend L., Prince B., Welch J., Cowan Z., Williamson R., Lyon C. (2017). A putative lysophosphatidylinositol receptor GPR55 modulates hippocampal synaptic plasticity. Hippocampus.

[141] Zhao Z., Cai Q., Xu Y. (2016). The lipidomic analyses in low and highly aggressive ovarian cancer cell lines. Lipids.

[142] Cannavo A., Liccardo D., Komici K., Corbi G., de Lucia C., Femminella G.D., Elia A., Bencivenga L., Ferrara N., Koch W.J. (2017). Sphingosine kinases and sphingosine 1-phosphate receptors: Signaling and actions in the cardiovascular system. Front. Pharmacol..

[143] Hatoum D., Haddadi N., Lin Y., Nassif N.T., McGowan E.M. (2017). Mammalian sphingosine kinase (SPHK) isoenzymes and isoform expression: Challenges for sphk as an oncotarget. Oncotarget.

[144] Rodriguez Y.I., Campos L.E., Castro M.G., Aladhami A., Oskeritzian C.A., Alvarez S.E. (2016). Sphingosine-1 phosphate: A new modulator of immune plasticity in the tumor microenvironment. Front. Oncol..

[145] Nagahashi M., Takabe K., Terracina K.P., Soma D., Hirose Y., Kobayashi T., Matsuda Y., Wakai T. (2014). Sphingosine-1-phosphate transporters as targets for cancer therapy. Biomed. Res. Int..

[146] Kunkel G.T., Maceyka M., Milstien S., Spiegel S. (2013). Targeting the sphingosine-1-phosphate axis in cancer, inflammation and beyond. Nat. Rev. Drug Discov..

[147] Kostenis E. (2004). Novel clusters of receptors for sphingosine-1-phosphate, sphingosylphosphorylcholine, and (lyso)-phosphatidic acid: New receptors for “old” ligands. J. Cell. Biochem..

[148] Kang Y.C., Kim K.M., Lee K.S., Namkoong S., Lee S.J., Han J.A., Jeoung D., Ha K.S., Kwon Y.G., Kim Y.M. (2004). Serum bioactive lysophospholipids prevent trail-induced apoptosis via PI3K/AKT-dependent cflip expression and bad phosphorylation. Cell Death Differ..

[149] Park K.S., Kim M.K., Lee H.Y., Kim S.D., Lee S.Y., Kim J.M., Ryu S.H., Bae Y.S. (2007). S1p stimulates chemotactic migration and invasion in ovcar3 ovarian cancer cells. Biochem. Biophys Res. Commun..

[150] Dai L., Xia P., Di W. (2014). Sphingosine 1-phosphate: A potential molecular target for ovarian cancer therapy?. Cancer Investig..

[151] Jin L., Liu W.R., Tian M.X., Fan J., Shi Y.H. (2016). The SPHKS/S1P/S1PR1 axis in immunity and cancer: More ore to be mined. World J. Surg. Oncol..

[152] Hong G., Baudhuin L.M., Xu Y. (1999). Sphingosine-1-phosphate modulates growth and adhesion of ovarian cancer cells. FEBS Lett..

[153] Baudhuin L.M., Jiang Y., Zaslavsky A., Ishii I., Chun J., Xu Y. (2004). S1p3-mediated AKT activation and cross-talk with platelet-derived growth factor receptor (PDGFR). FASEB J..

[154] Wang D., Zhao Z., Caperell-Grant A., Yang G., Mok S.C., Liu J., Bigsby R.M., Xu Y. (2008). S1p differentially regulates migration of human ovarian cancer and human ovarian surface epithelial cells. Mol. Cancer Ther..

[155] Devine K.M., Smicun Y., Hope J.M., Fishman D.A. (2008). S1p induced changes in epithelial ovarian cancer proteolysis, invasion, and attachment are mediated by GI and RAC. Gynecol. Oncol..

[156] Smicun Y., Gil O., Devine K., Fishman D.A. (2007). S1p and LPA have an attachment-dependent regulatory effect on invasion of epithelial ovarian cancer cells. Gynecol. Oncol..

[157] Smicun Y., Reierstad S., Wang F.Q., Lee C., Fishman D.A. (2006). S1p regulation of ovarian carcinoma invasiveness. Gynecol. Oncol..

[158] Troupiotis-Tsailaki A., Zachmann J., Gonzalez-Gil I., Gonzalez A., Ortega-Gutierrez S., Lopez-Rodriguez M.L., Pardo L., Govaerts C. (2017). Ligand chain length drives activation of lipid g protein-coupled receptors. Sci. Rep..

[159] Dai L., Liu Y., Xie L., Wu X., Qiu L., Di W. (2017). Sphingosine kinase 1/sphingosine-1-phosphate (S1p)/S1p receptor axis is involved in ovarian cancer angiogenesis. Oncotarget.

[160] Fyrst H., Saba J.D. (2010). An update on sphingosine-1-phosphate and other sphingolipid mediators. Nat. Chem. Biol..

[161] Illuzzi G., Bernacchioni C., Aureli M., Prioni S., Frera G., Donati C., Valsecchi M., Chigorno V., Bruni P., Sonnino S. (2010). Sphingosine kinase mediates resistance to the synthetic retinoid *N*-(4-hydroxyphenyl)retinamide in human ovarian cancer cells. J. Biol. Chem..

[162] Snider A.J., Orr Gandy K.A., Obeid L.M. (2010). Sphingosine kinase: Role in regulation of bioactive sphingolipid mediators in inflammation. Biochimie.

[163] Hait N.C., Allegood J., Maceyka M., Strub G.M., Harikumar K.B., Singh S.K., Luo C., Marmorstein R., Kordula T., Milstien S. (2009). Regulation of histone acetylation in the nucleus by sphingosine-1-phosphate. Science.

[164] Patmanathan S.N., Wang W., Yap L.F., Herr D.R., Paterson I.C. (2017). Mechanisms of sphingosine 1-phosphate receptor signalling in cancer. Cell Signal..

[165] Fan Q., Cheng Y., Chang H.M., Deguchi M., Hsueh A.J., Leung P.C.K. (2017). Sphingosine-1-phosphate promotes ovarian cancer cell proliferation by disrupting hippo signaling. Oncotarget.

[166] Wen Z., Zhao S., Liu S., Liu Y., Li X., Li S. (2015). MicroRNA-148a inhibits migration and invasion of ovarian cancer cells via targeting sphingosine-1-phosphate receptor 1. Mol. Med. Rep..

[167] Du W., Takuwa N., Yoshioka K., Okamoto Y., Gonda K., Sugihara K., Fukamizu A., Asano M., Takuwa Y. (2010). S1p(2), the G protein-coupled receptor for sphingosine-1-phosphate, negatively regulates tumor angiogenesis and tumor growth in vivo in mice. Cancer Res..

[168] Dai L., Liu Y.X., Xie L., Di W. (2018). Effect of S1PR2 inhibition on epithelial ovarian cancer SKOV3 cell proliferation in vitro and in vivo. Zhonghua Fu Chan Ke Za Zhi.

[169] Michaud J., Im D.S., Hla T. (2010). Inhibitory role of sphingosine 1-phosphate receptor 2 in macrophage recruitment during inflammation. J. Immunol..

[170] Goetzl E.J., Dolezalova H., Kong Y., Hu Y.L., Jaffe R.B., Kalli K.R., Conover C.A. (1999). Distinctive expression and functions of the type 4 endothelial differentiation gene-encoded G protein-coupled receptor for lysophosphatidic acid in ovarian cancer. Cancer Res..

[171] Argraves K.M., Wilkerson B.A., Argraves W.S. (2010). Sphingosine-1-phosphate signaling in vasculogenesis and angiogenesis. World J. Biol. Chem..

[172] Lucke S., Levkau B. (2010). Endothelial functions of sphingosine-1-phosphate. Cell. Physiol. Biochem..

[173] Kim K.S., Ren J., Jiang Y., Ebrahem Q., Tipps R., Cristina K., Xiao Y.J., Qiao J., Taylor K.L., Lum H. (2005). GPR4 plays a critical role in endothelial cell function and mediates the effects of sphingosylphosphorylcholine. FASEB J..

[174] Beach J.A., Aspuria P.J., Cheon D.J., Lawrenson K., Agadjanian H., Walsh C.S., Karlan B.Y., Orsulic S. (2016). Sphingosine kinase 1 is required for TGF-beta mediated fibroblastto- myofibroblast differentiation in ovarian cancer. Oncotarget.

[175] Shida D., Takabe K., Kapitonov D., Milstien S., Spiegel S. (2008). Targeting SPHK1 as a new strategy against cancer. Curr. Drug Targets.

[176] Visentin B., Vekich J.A., Sibbald B.J., Cavalli A.L., Moreno K.M., Matteo R.G., Garland W.A., Lu Y., Yu S., Hall H.S. (2006). Validation of an anti-sphingosine-1-phosphate antibody as a potential therapeutic in reducing growth, invasion, and angiogenesis in multiple tumor lineages. Cancer Cell.

[177] Siegel R.L., Miller K.D., Jemal A. (2018). Cancer statistics, 2018. CA Cancer J. Clin..

[178] Domcke S., Sinha R., Levine D.A., Sander C., Schultz N. (2013). Evaluating cell lines as tumour models by comparison of genomic profiles. Nat. Commun..

[179] Beaufort C.M., Helmijr J.C., Piskorz A.M., Hoogstraat M., Ruigrok-Ritstier K., Besselink N., Murtaza M., van I.W.F., Heine A.A., Smid M. (2014). Ovarian cancer cell line panel (OCCP): Clinical importance of in vitro morphological subtypes. PLoS ONE.

[180] Lee H.Y., Shin E.H., Bae Y.S. (2006). Sphingosylphosphorylcholine stimulates human monocyte-derived dendritic cell chemotaxis. Acta Pharmacol. Sin..

[181] Ceballos A., Sabatte J., Nahmod K., Martinez D., Salamone G., Vermeulen M., Maggini J., Salomon H., Geffner J. (2007). Sphingosylphosphorylcholine activates dendritic cells, stimulating the production of interleukin-12. Immunology.

